# Molecular Mechanisms Underlying Flax (*Linum usitatissimum* L.) Tolerance to Cadmium: A Case Study of Proteome and Metabolome of Four Different Flax Genotypes

**DOI:** 10.3390/plants11212931

**Published:** 2022-10-31

**Authors:** Veronika Berková, Miroslav Berka, Miroslav Griga, Romana Kopecká, Miroslava Prokopová, Markéta Luklová, Jiří Horáček, Iva Smýkalová, Petr Čičmanec, Jan Novák, Břetislav Brzobohatý, Martin Černý

**Affiliations:** 1 Department of Molecular Biology and Radiobiology, Faculty of AgriSciences, Mendel University in Brno, 61300 Brno, Czech Republic; 2 Plant Biotechnology Department, Agritec Plant Research, Ltd., 78701 Šumperk, Czech Republic

**Keywords:** heavy metals, toxicity, Cd^2+^, proteome, phenolic compounds, pipecolinic acid, HSP70, polyamines

## Abstract

Cadmium is one of the most toxic heavy metal pollutants, and its accumulation in the soil is harmful to agriculture. Plants have a higher cadmium tolerance than animals, and some species can be used for phytoremediation. Flax (*Linum usitatissimum* L.) can accumulate high amounts of cadmium, but the molecular mechanism behind its tolerance is unknown. Here, we employed four genotypes representing two fiber cultivars, an oilseed breeding line, and a transgenic line overexpressing the metallothionein domain for improved cadmium tolerance. We analyzed the proteome of suspensions and the proteome and metabolome of seedling roots in response to cadmium. We identified more than 1400 differentially abundant proteins representing putative mechanisms in cadmium tolerance, including metal-binding proteins and transporters, enzymes of flavonoid, jasmonate, polyamine, glutathione metabolism, and HSP70 proteins. Our data indicated the role of the phytohormone cytokinin in the observed responses. The metabolome profiling found that pipecolinic acid could be a part of the cadmium accumulation mechanism, and the observed accumulation of putrescine, coumaric acid, cinnamic acid, and coutaric acid confirmed the role of polyamines and flavonoids in tolerance to cadmium. In conclusion, our data provide new insight into cadmium tolerance and prospective targets for improving cadmium tolerance in other plants.

## 1. Introduction

Anthropogenic activities lead to heavy metal contamination. Industrialization, the application of phosphate fertilizers, municipal waste disposal, mining, coal-burning power, and inappropriate agricultural practices are some of the many factors adversely affecting the global environment and crop production [1,2,3,4]. Among heavy metals, cadmium is recognized as highly toxic to living organisms, and it is the third most dangerous after mercury and lead. It is a widespread toxic pollutant that is not an essential or beneficial element, and it has biological activity in terrestrial and aquatic organisms. Human activity has been estimated to release approximately 13,000 tons of cadmium per year that is eventually deposited in the soil, taken up by plants, and introduced into the food chain [5,6,7].

Plants are sessile organisms that cannot escape their habitat and have to compete for their environment. Therefore, plants are generally more tolerant of cadmium than other organisms. Cadmium interferes with the uptake of mineral nutrients such as calcium, potassium, copper, iron, and silicon, and has an impact on phosphorus, sulfur, and nitrogen assimilation [8,9]. A part of cadmium toxicity is its ability to bind thiol groups and disrupt the structure and activity of proteins [10]. The threshold of phytotoxic concentration varies between species and genotypes, but at toxic concentrations, cadmium inhibits root and stem elongation by reducing mitotic division [11,12] and induces severe damage to shoots, including chlorosis, necrotic lesions, wilting or leaf deformation [13].

Flax (*Linum usitatissimum* L.) belongs to the *Linaceae* family and is cultivated worldwide to produce oil, fiber, and various food. It is a well-known industrial crop for the production of clothing, linen bags, printing inks, and natural linoleum [14]. Flax plants also produce secoisolariciresinol diglucoside, a lignan that can chelate heavy metals [15]. Flax has been shown to tolerate cadmium and is suitable for the phytoremediation of contaminated soils [16,17,18]. Flax does not accumulate cadmium in ranges of 10,000 mg Cd^2+^ kg^−1^ like the cadmium hyperaccumulator species (e.g., *Thlaspi* spp. [19]), but it survives significantly higher concentrations of cadmium than other plants and has a suitable capacity for phytoremediation [18]. Interestingly, our understanding of molecular mechanisms of flax tolerance to cadmium is sparse, and previous proteome analysis revealed only 14 cadmium-responsive proteins [20]. Here, two cultivars (Jitka, Tábor), a breeding line (AGT), and one transgenic line expressing cadmium-binding protein metallothionein (MT) were grown in the presence of cadmium. The cadmium effect was followed in suspension cultures and roots of six-day-old seedlings. The results showed limitations of suspension culture experiments and demonstrated the role of HSP70 proteins, phytohormones, polyamines, phenolics, and sulfur metabolism in flax tolerance to cadmium.

## 2. Results

### 2.1. The Effects of Cadmium on Cell Viability and Root Growth

**Figure 1:**
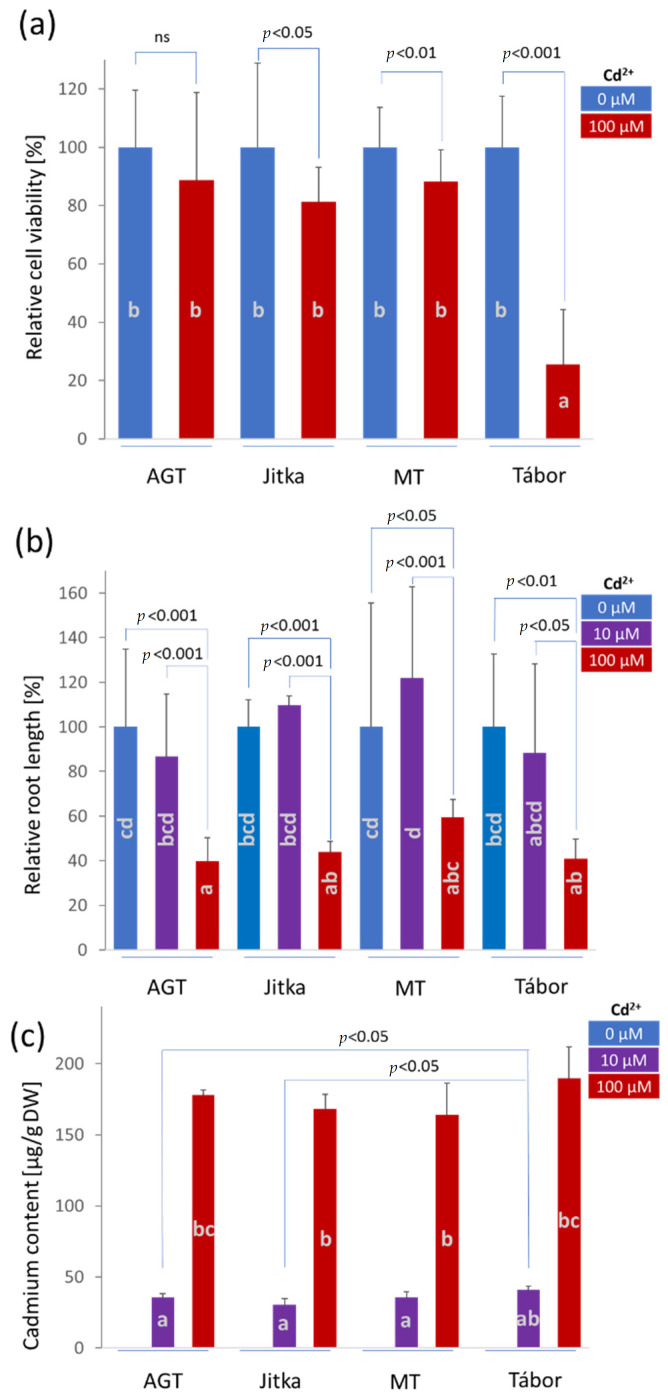
Cadmium tolerance in four genotypes employed in the study. (**a**) Relative cell viability of suspension cultures, (**b**) root length, and (**c**) cadmium content in shoots of six-day-old flax seedlings. The plots represent the means and standard deviation of at least five (**a**,**b**) and three (**c**) biological replicates. Different letters indicate significant differences (Kruskal-Wallis test, *p* < 0.05); *p*-values represent results of pair-wised comparisons and Student’s *t*-test.

Four genotypes were used in the experiment: the Jitka and Tábor fiber breeding cultivars, the AGT oilseed breeding line, and the MT transgenic line constitutively expressing the α-domain of mammalian metallothionein. First, the suspension cultures derived from these plants were exposed to 100 µM Cd^2+^, and cell viability was monitored as described in the materials and methods. Cultivar AGT showed lower viability, but the difference was not statistically significant (Figure 1a). Suspension cultures derived from the Jitka and MT transgenic lines showed a mild tolerance with an average decrease in viability of 19 and 12%, respectively. The lowest tolerance was found for the Tábor cultivar, which is in agreement with previously published reports [20]. Next, the tolerance to cadmium was evaluated by measuring the inhibition of root growth of flax seedlings (Figure 1b). The results of a two-way ANOVA confirmed the significant effect of genotype (*p* = 0.045) and cadmium concentration (*p* = 2.7 × 10^−12^) but did not show any statistically significant interaction between these two factors (*p* = 0.65). All plants showed tolerance to 10 µM Cd^2+^ with the cultivar Jitka and the transgenic line MT showing a relatively high but statistically insignificant increase in root length compared to respective mock-treated controls. Interestingly, the response to 100 µM Cd^2+^ was not significantly different between the tested genotypes (Kruskal-Wallis test, *p* < 0.05), and analysis of cadmium accumulation in shoots confirmed this similarity (Figure 1c). The shoots of seedlings grown in the presence of 10 µM Cd^2+^ contained cadmium at an average concentration of 35.7 ± 3.7 µg/g (dry weight). The accumulation at 100 µM Cd^2+^ was significantly higher, resulting in an average cadmium concentration of 175.0 ± 9.9 µg/g (dry weight). The cadmium accumulation was slightly higher in the Tábor cultivar showing concentration values 14.5 and 8.5% higher than the mean concentration in the genotypes tested at 10 and 100 µM Cd^2+^, respectively. However, a two-way ANOVA analysis showed that only the concentration of cadmium in the medium had a significant effect (*p* < 0.05). The genotype effect was not significant (*p* > 0.2), and there was no significant interaction between the two experimental factors (*p* > 0.55).

### 2.2. Proteome Analysis of Flax Suspension Cultures

**Figure 2:**
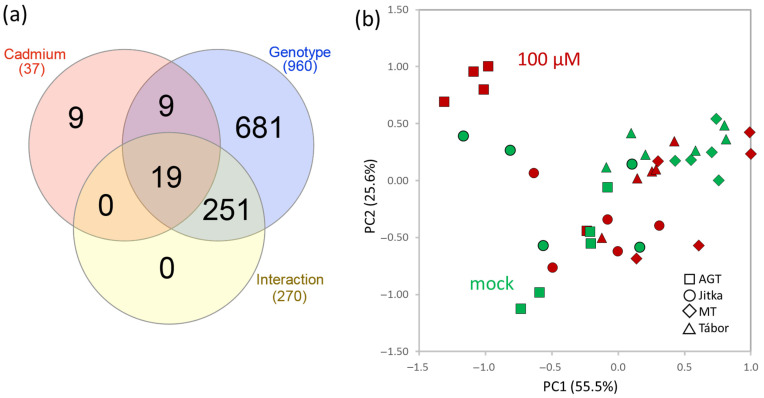
The proteome profile of the flax suspension cultures did not show the expected separation of the mock and cadmium treated cells. (**a**) Results of two-way ANOVA analysis visualized in a Venn diagram and (**b**) PCA based on profile of all 969 differentially abundant proteins. For details, see Supplementary Tables S1 and S2.

**Figure 3:**
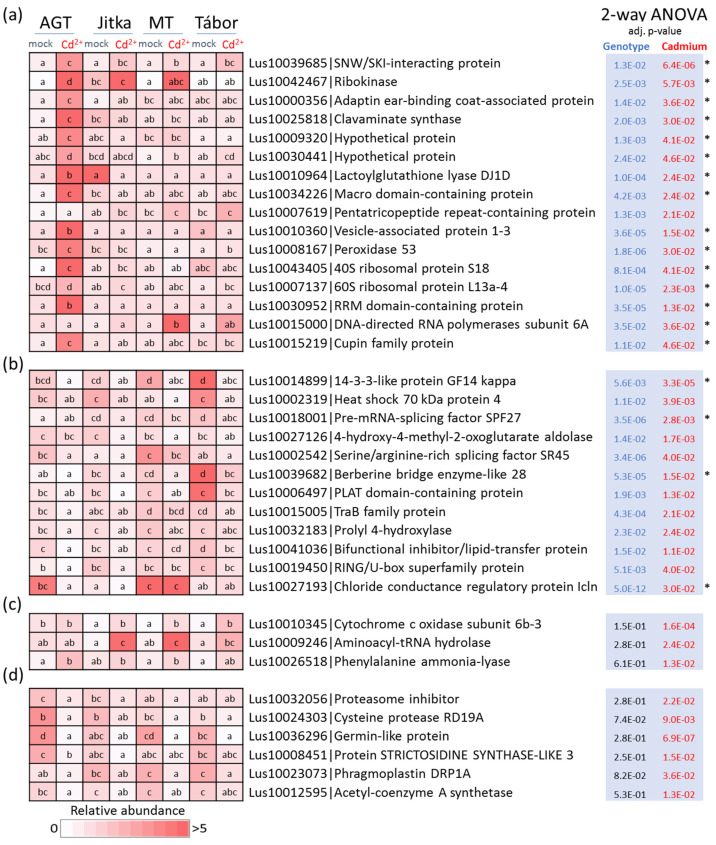
Cadmium-responsive proteins identified in flax suspension cultures. (**a**,**b**) Genotype-specific effect on protein abundance combined with (**a**) the cadmium-induced accumulation and (**b**) a decrease in abundance. (**c**,**d**) Genotype-independent effects of cadmium on protein accumulation (**c**) and decrease in abundance (**d**). Heat maps represent the mean relative abundances of five biological replicates; letters represent the results of Kruskal-Wallis and Dunn’s test (*p* < 0.05); blue, significant effects of genotype; red, significant effects of cadmium; asterisks represent significant interaction between genotype and cadmium concentration.

The proteome analysis of suspension culture extracts provided identification and quantitation of 4178 and 2311 proteins, respectively. An ANOVA analysis found more than 900 significant differences, but most of these were related to genotype (Figure 2a). Accordingly, the corresponding principal component analysis (PCA) showed a similarity between the response to cadmium and mock-treated controls (Figure 2b). Only 37 differentially abundant proteins were cadmium-responsive at *p* < 0.05 (Figure 3).

The proteins of interest that were accumulated in response to cadmium (Figure 3a,c) included a key enzyme in the biosynthesis of phenolic compounds (Phenylalanine ammonia-lyase), oxidative stress response enzymes (Lactoylglutathione lyase DJ1D, Peroxidase), two ribosomal proteins, proteins of RNA metabolism (a subunit of RNA polymerase and an ortholog of the splicing factor SNW/SKI-interacting protein), an enzyme of tryptophan and histidine biosynthesis (Ribokinase), cupin family storage protein, an ortholog of clavaminate synthase (previously found in response to cadmium in earthworm; [21]), and proteins involved in vesicle transport and endocytosis (Adaptin ear-binding coat-associated protein, Vesicle-associated protein 1-3). A decrease in abundance was found for 18 proteins, including a 14-3-3 protein (signaling), strictosidine synthase (produces the key intermediate of indole alkaloid biosynthesis), the HSP70 family protein, an ortholog of the berberine bridge enzyme (mutant sensitive to salt stress; [22]), an enzyme that cleaves 4-hydroxy-4-methyl-2-oxoglutarate into pyruvate, PLAT domain protein with a putative role in tolerance to abiotic stress [23], a defense-related protein germin, acetyl-CoA synthetase, and two putative splicing factors (SPF27 and SR45). However, the most interesting cadmium-responsive proteins on the list of proteins with a decrease in abundance (Figure 3b,d) were orthologs of proteins involved in cell expansion, namely chloride conductance regulatory protein ICln that participates in the regulation of cell swelling [24] and microtubule-associated protein Phragmoplastin (involved in vesicular trafficking; the mutant shows defects in cell expansion; [25]).

### 2.3. Flax Root Proteome in Response to Cadmium

**Figure 4:**
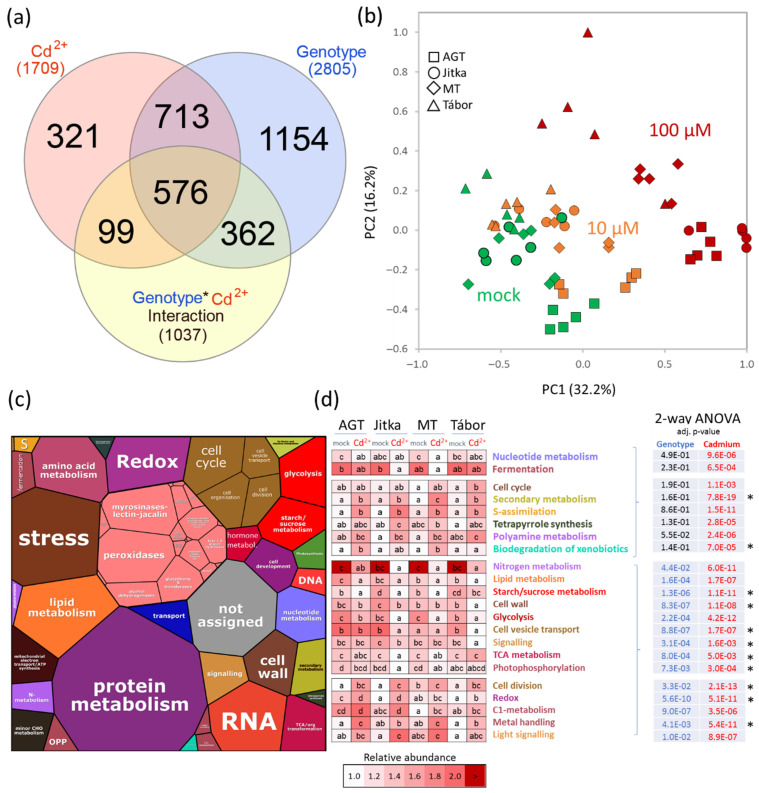
Cadmium response in root tissue of four different genotypes. Comparison of mock-treated roots and response to 100 µM Cd^2+^. (**a**) Results of the two-way ANOVA analysis visualized in a Venn diagram, (**b**) PCA based on the profile of 1435 differentially abundant proteins (*p* < 0.05, at least 1.5-fold change), (**c**) visualization of functional categories in the ProteoMap, and (**d**) significant differences visualized on a heat map. The ProteoMap corresponds to the estimated content in the mock-treated AGT plants. Asterisks indicate a significant interaction of genotype and cadmium concentration on protein abundances. The letters represent significant differences (*p* < 0.05, ANOVA, Tukey’s HSD). For details, see Supplementary Table S3.

Proteome analysis of root tissue extracts provided identification and quantitation for 6237 and 4467 proteins, respectively (Table S5). An ANOVA analysis found 3225 significant differences, and more than half of these were significant responses to cadmium (Figure 4a). After applying a 1.5-fold threshold filter, 1435 differentially abundant cadmium-responsive proteins were selected for further analyses, representing 26% of the estimated protein content in the mock-treated AGT plants. The resulting PCA clearly separated the proteomes of mock- and cadmium-treated plants (Figure 4b). The proteome composition was analyzed by ProteoMaps using annotations of the corresponding *Arabidopsis* orthologs (Figure 4c). The consecutive comparison highlighted a statistically significant (ANOVA, *p* < 0.05) increase in the abundance of secondary metabolism enzymes, including phenylpropanoid biosynthesis (caffeoyl-CoA O-methyltransferase Lus10014074, cinnamoyl-CoA reductase Lus10041651, and melatonin biosynthetic enzyme acetylserotonin O-methyltransferase), flavonoids (phenylcoumaran benzylic ether reductase Lus10042313, UDP-glycosyltransferase 72B1 Lus10005951), and isoprenoids (solanesyl diphosphate synthase, heterodimeric geranylgeranyl pyrophosphate synthases Lus10028508 and Lus10016803, alpha-humulene/(-)-(E)-beta-caryophyllene synthase Lus10031590, and violaxanthin de-epoxidase Lus10021986). Cadmium treatment also induced the accumulation of sulfur assimilation enzymes (sulfite reductase Lus10030131, 5′-adenylylsulfate reductase Lus10020040, ATP sulfurylase Lus10033158), polyamine biosynthesis (spermidine synthases Lus10030629, Lus10012996), metal handling (selenium-binding protein Lus10037336, aluminum-induced protein Lus10034038, copper transport protein Lus10043444), and redox metabolism (family of protein disulfide isomerases, GSH biosynthetic enzymes Lus10002001 and Lus10029976, GSH reductase Lus10016798, 5-oxoprolinase Lus10024411, and peroxidases). The analysis also indicated an increase in the abundance of proteins modulating cell division (cell division control proteins Lus10021442, Lus10021441, Lus10016123; plastid division FtsZ Lus10021367; mitochondria and peroxisomal fission protein Lus10036023; component of the anaphase promoting complex Lus10006853). A significant decrease was found for 11 categories, including nitrogen metabolism (glutamine synthetase Lus10004037), lipid metabolism (at least ten enzymes of fatty acid synthesis and lipid degradation), the cell wall metabolic processes (Fasciclin-like proteins, xyloglucan endotransglucosylase/hydrolases, expansin Lus10003336, pectinesterase Lus10008203, pectin acetylesterase Lus10007200, endoglycosidase Lus10008823, xylosidase Lus10016858, fucose synthase Lus10037102, UDP-glucuronic acid decarboxylase Lus10005450), and carbohydrate-active enzymes (CAZymes; nine and 13 enzymes of glycolysis and starch/sucrose metabolism, respectively). Both PCA and functional analyses confirmed the similarity in response to 100 µM cadmium in all tested genotypes, but some responses seemed to be attenuated in Tábor (found sensitive in a suspension culture viability assay, Figure 1a), including biodegradation of xenobiotics, C1-metabolism, and starch/sucrose metabolism (Figure 4d).

### 2.4. Comparison of Suspension Culture and Root Tissue Response to Cadmium

Interestingly, almost all cadmium-responsive proteins identified in the suspension culture experiment were found in the root proteome. However, some responses were only genotype-specific, and only eight showed a similar statistically significant response to 100 µM Cd^2+^, including 60S ribosomal protein Lus10007137, germin Lus10036296, and peroxidase Lus10008167 (Table S3). Five of the shared cadmium-responsive proteins in root proteome showed a similarity at lower cadmium concentration (10 µM), including Acetyl-coenzyme A synthetase (Lus10012595), Heat Shock Protein 70 (HSP70, Lus10002319), and proteasome inhibitor Lus10032056. Phenylalanine ammonia-lyase (Lus10026518) and Pentatricopeptide repeat-containing protein (Lus10007619) showed a similar trend, but the response in the root proteome was not statistically significant. Finally, at least ten root proteins showed a contrasting response to that found in suspension cultures, indicating a lower transferability between the suspension culture results and an organ response of the complex organism.

### 2.5. Metabolome Profiling

**Figure 5:**
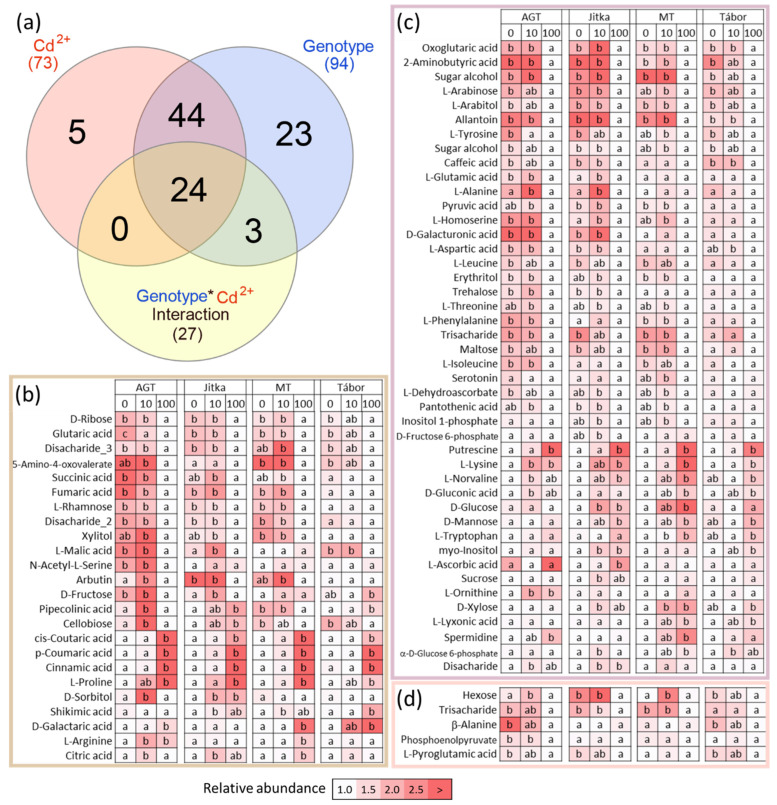
Metabolome analysis of flax root in response to cadmium. (**a**) Results of two-way ANOVA analysis visualized in a Venn diagram and the corresponding heat map visualization of subsets of (**b**) 24 metabolites whose abundances were affected by cadmium and genotype with significant interaction of these two factors, (**c**) 44 metabolites that showed genotype- and cadmium- specific responses and no interaction of these two factors, and (**d**) five metabolites that showed only a genotype-independent response to cadmium. Different letters indicate significant differences (Kruskal-Wallis and Dunn’s test, *p* < 0.05). For simplicity, the tests and normalization in the heat maps are limited to the genotype level. For cross-genotype comparisons, see the Supplementary Table S6.

A GC-MS metabolome analysis of root tissue provided quantitative data for 100 polar and semipolar metabolites. In contrast to proteomics data, most metabolites showed genotype-specific accumulation patterns. Only five metabolites had a genotype-independent cadmium-specific response, namely phosphoenolpyruvate, pyroglutamate, β-alanine, and two unidentified carbohydrates (Figure 5a–d). The cadmium effect was most pronounced at a high concentration and had a predominantly negative effect on the abundances of identified differentially abundant metabolites. Based on the Kruskal-Wallis test, cadmium treatment induced a significant decrease in the abundance of 28 metabolites in at least three genotypes. An additional 15 metabolites were depleted but did not pass the significance threshold (*p* < 0.05, Kruskal-Wallis). An increase in abundance was found only for 13 metabolites, six showed a higher abundance below the significance threshold, and 11 showed genotype- and concentration-specific responses, including glycoside arbutin. This secondary metabolite was accumulated in response to 10 µM cadmium and only in three genotypes that did not show any cadmium toxicity response in suspension cultures, indicating its putative role in cadmium tolerance. The pathway impact analysis based on identified differentially abundant metabolites showed a decrease in the citrate cycle and pyruvate metabolism and an increase in secondary metabolites. A negative impact was found on multiple amino acid metabolism pathways (alanine, aspartate, glutamate, tyrosine, glycine, serine, threonine, valine, leucine, and isoleucine), but the abundance of stress-responsive amino acid proline was significantly increased. Polyamines putrescine and spermidine accumulated in all four genotypes, and polyamine precursor arginine was significantly more abundant in genotypes AGT and MT.

### 2.6. Integrative Analysis of Proteome and Metabolome

**Figure 6:**
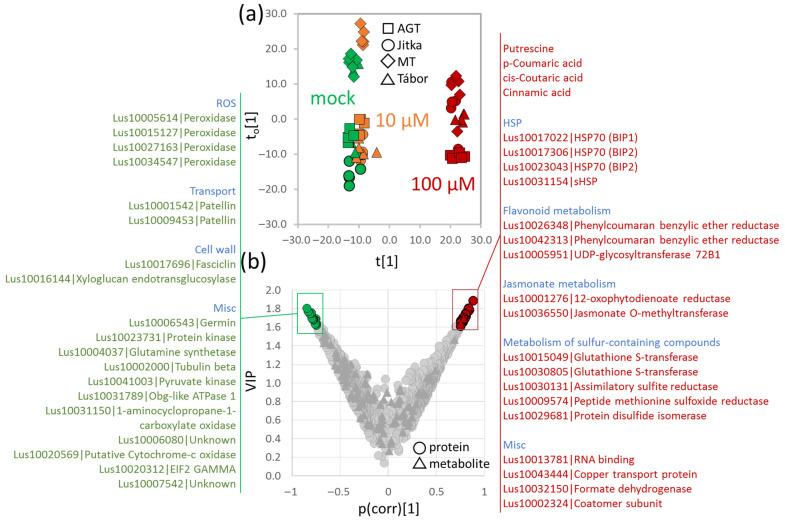
Identification of proteins and metabolites that show significant correlation with cadmium effects on the molecular composition of roots. Orthogonal partial least squares discriminant analysis (**a**) followed by VIP (variable importance in projection); (**b**) Identified proteins and metabolites with significant correlation (absolute threshold 0.75) are listed. For details, see Supplementary Tables S4 and S5.

Next, to identify compounds with a putative role in cadmium response, the correlation between the cadmium concentration in the medium and the molecular composition of roots was analyzed via an orthogonal partial least squares (OPLS) regression analysis (Figure 6a). That pinpointed a set of 41 molecules that correlated with the cadmium response, including 37 proteins and four metabolites (Figure 6b, Supplementary Materials Tables S4 and S5). The set of 18 proteins that accumulated in response to cadmium included a family of HSP70 proteins and one small HSP, three enzymes of flavonoid metabolism, two enzymes of jasmonic acid/oxylipin metabolism, five enzymes of sulfur-compound metabolism, a copper transporter, an RNA binding protein, formate dehydrogenase, and protein involved in vesicle transport from the endoplasmic reticulum. The set proteins that were less abundant in response to cadmium included four peroxidases, two isoforms of carrier protein patellin, two proteins involved in cell wall metabolism, and 11 proteins of different functions (Figure 6b). Metabolites were found only in the list of compounds with positive correlation and included polyamine putrescine and three phenolic compounds (coumaric acid, coutaric acid, and cinnamic acid).

**Figure 7:**
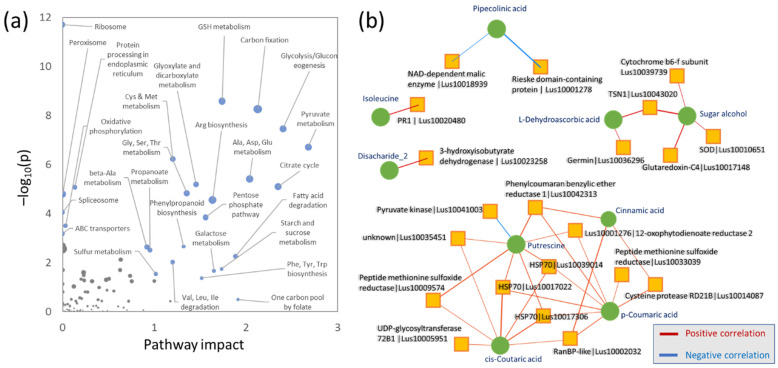
Integrative analysis of proteomics and metabolomics data. (**a**) Visualization of metabolic pathways significantly impacted by cadmium treatment and (**b**) significant intra-omics correlations. The pathway impact analysis was visualized by MetaboAnalyst. Omics data interaction was evaluated by OmicsAnalyst using Pearson’s correlation threshold of 0.75 for intra-omics interactions. For details, see Supplementary Tables S4 and S5.

The pathway impact analysis highlighted the role of glutathione metabolism, carbon fixation and energy metabolism, including pyruvate metabolism, glycolysis, and the citric acid cycle. It also indicated the role of amino acid metabolism, phenylpropanoid metabolism, and sulfur metabolism (Figure 7a). A significant enrichment was also found for ribosomal proteins, ABC transporters, and proteins involved in protein processing, but none of these categories had an impact on the identified metabolic pathways. Finally, metabolome and proteome profiles were compared, and relationships between key features were assessed by Pearson’s correlation. In total, five clusters formed by nine metabolites and 21 proteins were revealed (Figure 7b). Unsurprisingly, the largest cluster showed similarity to the OPLS/VIP analysis results and pinpointed the relationship between proteins of the HSP70 family, putrescine, and phenolic compounds (coumaric acid, cinnamic acid, and coutaric acid). This cluster also contained other proteins found on the list (peptide methionine sulfoxide reductase, glycosyltransferase, enzymes of jasmonic acid biosynthesis and flavonoid metabolism), an unknown protein (Lus10035451), cysteine protease (Lus10014087), RanBP-like protein (putative role in nuclear transport), and pyruvate kinase (Lus10041003). The second largest cluster was formed by dehydroascorbate and proteins involved in ROS metabolism (superoxide dismutase, glutaredoxin). It also included an unidentified sugar alcohol, an ortholog of cytoprotective ribonuclease TSN required for resistance to abiotic stresses [26], and a subunit of cytochrome b6-f complex that contributes to the thermal dissipation of light energy and resistance to photo-oxidative damage. The last cluster of interest is formed by pipecolinic acid. This metabolite showed a highly genotype-specific accumulation pattern, with the highest content in Tábor (4-10-fold higher abundance than other genotypes; Table S6), and its abundance showed a negative correlation with NAD-dependent malic enzyme and an unknown protein containing the 2Fe-2S Rieske domain.

## 3. Discussion

### 3.1. Cadmium Response in Four Different Genotypes

Four genotypes were employed in the described analyses. Two fiber cultivars used in the previous proteomics experiments (Tábor, Jitka [20]) were complemented by a transgenic line expressing the gene coding metallothionein domain and the corresponding parental line AGT. The suspension culture experiment confirmed the previous observation and a lower cadmium tolerance of Tábor. However, no significant differences were found between the transgenic line and the other two genotypes (Figure 1a). The consecutive proteome analysis showed genotype-specific differences, but the number of identified cadmium-responsive proteins was surprisingly low (Figure 2a,b). The response of six-day-old seedlings did not show marked differences in cadmium tolerance (Figure 1b). All genotypes manifested attenuated growth, but viability was not compromised. The comparison of proteome and metabolome showed both a surprising diversity and similarity in the identified differentially abundant proteins and metabolites. The identified cadmium-responsive pathways were mostly similar, but the modulations of individual proteins and metabolites were predominantly genotype-specific (Figure 4a and Figure 5a). For instance, only 21 metabolites showed a similar and statistically significant response to cadmium in all genotypes (Figure 5). Tábor showed the highest divergence among the root proteome datasets. In contrast to other genotypes, the roots of Tábor did not show significant differences in the estimated abundance of proteins for starch/sucrose metabolism and the TCA metabolism at 100 µM Cd^2+^(Figure 4d). However, all four genotypes accumulated an ortholog of selenium-binding protein (Lus10037336; binds cadmium and promotes cadmium tolerance [27]), aluminum-induced protein Lus10034038, copper transport protein (Lus10043444), and showed a decrease in abundance of ferritin (Lus10017433). That is well in line with the previously published effects of cadmium on copper and iron homeostasis [28,29], and the observed pattern correlates with a higher cadmium tolerance in flax.

### 3.2. Comparison of Cadmium-Responsive Proteins in Flax and Model Plant Arabidopsis Thaliana

The set of identified cadmium-responsive proteins was compared with the set of annotated cadmium-responsive proteins found in *Arabidopsis* (https://www.arabidopsis.org/, accessed on 4 October 2022, https://www.uniprot.org/, accessed on 4 October 2022). The databases contained more than 100 cadmium-responsive proteins, but the overlap with cadmium response in flax root tissue was low. Only 12 flax proteins representing orthologs of 11 *Arabidopsis* proteins were shared, including enzymes of cysteine and glutathione biosynthesis (Lus10015947, Lus10015947, Lus10002001), glutathione S-transferase (Lus10020735), ADP-ribosylation factor GTPase-activating protein (Lus10023766), alpha-xylosidase (Lus10034315), 60S ribosomal protein L4-1 (Lus10026476), ABC transporter (Lus10037562), copper transport protein CCH (Lus10043444), selenium binding protein (Lus10037336), uroporphyrinogen decarboxylase (Lus10011170), and Aldolase-type TIM barrel family protein (Lus10003271). These results indicate that the cadmium effect on metal transporters, glutathione metabolism, and ribosome composition is evolutionary conserved.

### 3.3. Cadmium Response in Suspension Cultures Could Correlate with a Higher Cadmium Import in Cultivar Tábor

Cultivars Jitka and Tábor were previously studied for cadmium uptake, accumulation, and tolerance [17,18,20,30,31]. Previous reports showed a contrasting response of the Tábor cultivar to cadmium. In the experiments with suspension cultures, this cultivar was found to be susceptible [20], but a study that compared 23 flax cultivars showed that Tábor had the highest tolerance to cadmium (EC_50_: 0.28 mM), 1.4-fold higher than cultivar Jitka [31]. Our data confirmed that the seemingly contradictory results were correct. Suspension cultures were more susceptible, but we did not find any significant differences in the cadmium tolerance of seedlings (Figure 1a,b). Furthermore, our results of cadmium content determination that could indicate the reason for the observed differences (Figure 1c). It seems that Tábor is accumulating more cadmium. The pairwise comparison showed that Tábor had a significantly higher cadmium concentration at 10 µM Cd^2+^ than Jitka or AGT (Figure 1c) and a higher concentration than the transgenic line MT (*p* < 0.1). It is thus likely that a higher accumulation in seedlings is accompanied by a promoted detoxification and cadmium sequestering in shoots, a process that does not operate under suspension culture conditions. The root proteome did not reveal any Tábor-specific differences in proteins associated with membrane transport (Table S4), but Tábor had the highest abundance (1.2-1.8-fold more) of the selenium binding protein Lus10037336 discussed above. The suspension culture proteome indicated one candidate that could be associated with a higher cadmium influx, namely PLAT domain-containing protein Lus10006497 (Figure 3b). The PLAT domain (Polycystin-1, Lipoxygenase, Alpha Toxin) is found in membrane-associated proteins, and PLAT domain proteins have been found in response to abiotic stressors, including temperature and salinity [23,32]. It is possible that this protein could play a direct or indirect role in cadmium transport. Besides the differences found in suspension cultures, roots of Tábor seedlings accumulated a significantly higher amount of pipecolinic acid. This metabolite is not only integral in acquired systemic resistance [33]. It is also an organic acid and a chelating agent capable of binding cadmium.

### 3.4. Polyamines, Jasmonic Acid, and Cytokinin Promoted Tolerance to Cadmium

The OPLS highlighted the role of phytohormones in response to cadmium, including polyamines, jasmonic acid (positive correlation), and ethylene (negative correlation). The abundance of two orthologs of polyamine biosynthetic enzymes was increased in response to cadmium, namely Lus10030629 (Spermidine synthase 2) and Lus10012996 (Spermidine synthase 1), and metabolome profiling showed an increase in spermidine and its precursor putrescine (Figure 5c). Polyamines are growth regulators that accumulate in response to cadmium [34], and previous reports indicate that polyamine treatment alleviates some effects of heavy metal toxicity [35,36,37]. Jasmonic acid was reported to suppress cadmium uptake and transport in *Arabidopsis*, and exogenous treatment with methyl jasmonate alleviated symptoms of cadmium stress [38]. Besides jasmonic acid and polyamine metabolism enzymes, the cadmium-responsive proteins included at least 15 additional enzymes of phytohormone metabolism (Table S4). The comparison of identified differentially abundant proteins with the database of previously identified phytohormone-responsive proteins [39] found the highest overlap for abscisic acid-responsive proteins (216 proteins), cytokinin-responsive proteins (179), and jasmonic acid-responsive proteins (112). Abscisic acid is a regulator of abiotic stress response reactions and its role in cadmium tolerance has been described [40,41]. Plant hormone cytokinin regulates root growth, and its synthesis is upregulated in response to heavy metals [42]. That is well in line with the observed response of identified cadmium-responsive proteins in the roots of flax. At least 75 proteins showed a response similar to the previously reported response to cytokinin, including enzymes of sulfur metabolism (cysteine synthases Lus10015947, Lus10025589, and Lus10019003; 5′-adenylylsulfate reductase Lus10020040) and three glutathione-S-transferases (Lus10037234, Lus10036688, Lus10030805). These enzymes are an integral part of cadmium detoxification by glutathione conjugation and were found to accumulate in the root proteome of plants treated with cadmium (Figure 6b). Cytokinin has previously been reported to induce an accumulation of glutathione S-transferases [43], and a recent study showed that the application of exogenous cytokinin significantly decreases the free glutathione pool [44]. This indicates that the role of cytokinin is not limited to the regulation of root growth, but it plays a more prominent role in the cadmium detoxification process.

### 3.5. HSP70 Proteins–Integral Components of Cadmium Tolerance?

Intra-omics analysis and OPLS/VIP identified proteins of the HSP70 family as key components of the response to cadmium. These multifaceted proteins play a role in protein folding, quality control, and protein transport and are involved in abiotic and biotic stress responses [45]. HSP70 has two calcium binding sites [46], and its accumulation could be correlated with the cadmium-calcium exchange. However, the role of HSP70 in cadmium tolerance has been implicated in a previous study of plant-growth-promoting bacteria that increased tolerance to cadmium in switchgrass [47], and a member of the HSP70 family was identified as a cadmium resistance loci in yeast [48]. Here, three cadmium-responsive HSP70 were found in flax roots, and all showed a high sequence similarity to *Arabidopsis* BIP proteins (localized in the nucleus and endoplasmic reticulum). BIPs are regulated by salicylic acid and play a role in the secretion of pathogenesis-related (PR) proteins [45]. It is thus likely that an accumulation of these HSP70 promotes defense mechanisms. Moreover, the overexpression of BIP reportedly conferred cadmium tolerance in transgenic tobacco [49] and mediated cadmium-induced autophagy [50]. A recently published study showed that cadmium triggered unfolded protein response in Arabidopsis thaliana and upregulated BIP3 [51]. These findings and results reported here indicate that HSP70s located in the endoplasmatic reticulum are integral components of cadmium tolerance.

### 3.6. Observed Decrease in Germin Proteins Could Indicate Reallocation of Resources to Abiotic Stress Response

The set of cadmium-responsive proteins of interest also contained protein germin Lus10036296. This differentially abundant protein was found in suspension cultures (a decrease in abundance, Figure 3d) and roots (correlating with dehydroascorbate, Figure 7b; a decrease in three genotypes). Five other germin proteins showed a decrease in abundance in response to cadmium in roots, including Lus10006543 (a negative correlation with cadmium concentration, Figure 6b). This protein family is vital for plant defense [52], and it has been shown that an increased abundance of germines promotes resistance against plant pathogens [53,54,55]. The observed response to cadmium thus likely represents a shift and reallocation of resources to an abiotic stress response.

### 3.7. Reactive Oxygen Species and Phenolics in Response to Cadmium

One of the cadmium toxicity effects in plants is manifested in the reactive oxygen species (ROS) production [56], and the plant antioxidant system has been shown to play a fundamental role in addressing the resulting oxidative stress [57,58]. The ROS-metabolism enzymes were significantly accumulated in response to cadmium (Figure 4d), and the observed accumulation of phenolic acids could also be a part of ROS scavenging. Some reports have indicated that phenolic compounds can remove hydroxyl radicals more efficiently than antioxidant enzymes [59]. Furthermore, phenolic acids can form complexes with heavy metals, and cinnamic and coumaric acids that were accumulated in response to cadmium (Figure 6b) were reportedly one of the most effective in combining with cadmium [59].

## 4. Materials and Methods

### 4.1. Plant Material

The *Linum usitatissimum* L. seeds were obtained from the Flax germplasm collection of Agritec Ltd., ŠUMPERK, Czech Republic. Genotypes used in the experiments represented two technological types: fiber flax–two Czech commercial cultivars, Jitka (bred and registered by Agritec Ltd., 1992) and Tábor (bred and registered by SEMO a.s., 2002); linseed–breeding line AGT 917 (breeder Dr. E. Tejklová, Agritec Ltd.) used as parental genotype for *Agrobacterium*-mediated transformation with an α-domain of mammalian metallothionein 1a (αMT1a); line MT [60]. For omics analyses, seeds were surface sterilized overnight using chlorine gas and aerated in a flow cabinet for 15 min. Seeds were pre-germinated on filter paper with a half-strength Murashige and Skoog medium for two days (8 mL of media per 80 seeds, 22 °C, 100 µmol m^−2^ s^−1^ photon flux density, 16/8 h light/dark cycle), and growing seedlings were transferred to Magenta boxes (Steri Vent Containers, Duchefa) containing 80 mL of a half-strength Murashige and Skoog medium solidified with 0.8% plant agar (Duchefa) and supplemented with the corresponding concentration of Cd(NO_3_)_2_. After four days of cultivation, the roots of six-day-old seedlings were separated and flash-frozen in liquid nitrogen. For all omics experiments, at least five biological replicates were collected. Plant material for cadmium determination was prepared under similar conditions, and shoots of at least 40 plants were collected per biological replicate.

Flax suspension cultures were established, and their viability was evaluated as described previously [20,61]. Briefly, callus was induced on hypocotyl segments (5 mm) of seven-day-old seedlings grown on modified Murashige and Skoog medium supplemented with 0.215 mg L^−1^ kinetin, 0.0225 mg L^−1^ 2,4-dichlorophenoxyacetic acid, 3% *w*/*v* sucrose, and 0.5% *w*/*v* agar, pH 5.8 (16 h photoperiod; 22 °C). The yellow-green friable callus obtained from each cultivar was mechanically disrupted, inoculated into 25 mL of modified liquid Murashige-Skoog medium (composition as described above), and incubated in the dark on a rotary shaker (100 rpm, 22 °C). Newly established suspensions were subcultured biweekly into a fresh medium to obtain stabilized, well-growing cultures. The viability of suspensions was monitored by Trypan Blue staining. The stabilized suspension cultures composed of single cells and cell aggregates/microcalli (ca four months after callus initiation) were treated with 100 µM Cd(NO_3_)_2_. After 72 h, the cultures were sampled for proteome analysis, and the viability of cells/cell clusters was recorded via TTC (2,3,5-triphenyltetrazolium) staining [20]. The image analysis was performed using NIS-Elements imaging software version 5.20.01 (LIM Prague, Praha, Czech Republic). The experiment was done in five biological replicates.

### 4.2. Cadmium Determination

Cadmium content was determined as described previously [17,62]. In brief, flax shoots were digested using a microwave oven system (ETHOS D, Milestone) with an energy output of 0–400 W. Approximately 250 mg of dry plant material was placed into the Teflon microwave digestion vessels and digested in a mixture of 5 mL of 65% HNO_3_ and 1 mL of 30% H_2_O_2_. The digested samples were diluted to a final volume of 25 mL with deionized water, and the cadmium content was determined by graphite furnace atomic absorption spectroscopy (SOLAAR M, Thermo Fisher Scientific) equipped with deuterium background correction, graphite furnace GF95, and a hollow cathode lamp. The wavelength used for cadmium quantification was 228.8 nm. The analysis was done in three biological replicates.

### 4.3. Proteome Analysis

Approximately 25 mg of homogenized tissue were extracted for omics analyses as described previously [63,64,65], and portions of samples corresponding to 5 µg of peptide were analyzed by nanoflow reverse-phase liquid chromatography-mass spectrometry using a 15 cm C18 Zorbax column (Agilent), a Dionex Ultimate 3000 RSLC nano-UPLC system, and the Orbitrap Fusion Lumos Tribrid Mass Spectrometer (Thermo Fisher Scientific). The measured spectra were recalibrated and searched against the *L. usitatissimum* v1.0 [66] and common contaminants databases using Proteome Discoverer 2.5 (Thermo Fisher Scientific). The quantitative differences were determined by Minora, employing precursor ion quantification followed by normalization (total area) and calculation of relative peptide/protein abundances. Protein functional annotations were obtained by searching protein sequences against Arabidopsis thaliana proteome using STRING 11.0 [67]. The annotation for proteins that did not match any *Arabidopsis* orthologs were found using UniProt database BLAST (https://www.uniprot.org/blast; accessed on 20 August 2022). The analysis was done in five biological replicates.

### 4.4. Metabolome Analysis

Metabolites were extracted with tert-butyl methyl ether:methanol:water mixture, and aliquots were derivatized and measured using a Q Exactive GC Orbitrap GC-tandem mass spectrometer and Trace 1300 Gas chromatograph (Thermo Fisher Scientific) [68,69]. Data were analyzed by Compound Discoverer 3.2 (Thermo Fisher Scientific) and searched against NIST2014, the GC-Orbitrap Metabolomics library, and the in-house library. Only metabolites fulfilling identification criteria (score ≥ 75 and ΔRI < 1.5%) were included in the list, and the final evaluation was done using manual inspection in Skyline [70]. The analysis was done in five biological replicates.

### 4.5. Statistics

The reported statistical tests were generated and implemented as follows using default and recommended settings unless otherwise indicated. The reliability of protein and metabolite identifications were assessed in Proteome Discoverer 2.5 (Thermo Fisher Scientific) and Compound Discoverer 3.2 (Thermo Fisher Scientific). The Student’s *t*-test was calculated using MS Excel. For ANOVA with Tukey’s HSD and Kruskal-Wallis tests, the Real Statistics Resource Pack software for MS Excel (Release 6.8; Copyright 2013–2020; Charles Zaiontz; www.real-statistics.com, accessed on 4 October 2022) and MetaboAnalyst 5.0 [71] were used. Intraomics interactions were analyzed by OmicsAnalyst [72] using Pearson’s correlation. PCAs were performed in MetaboAnalyst 5.0. OPLS and VIP were performed in SIMCA 14.1 (Sartorius). Significant differences refer to *p* < 0.05, unless otherwise stated.

## Data Availability

Data supporting the results are provided in the tables in the Supplementary Materials.

## Supplementary Materials

The following supporting information can be downloaded at: https://www.mdpi.com/article/10.3390/plants11212931/s1, Table S1: ANOVA analysis of protein abundances in response to cadmium—suspension cultures title; Table S2: Differentially abundant proteins in response to cadmium treatment—suspension cultures; Table S3: Root proteome analysis—supplementary material to Figure 4d; Table S4: Root proteome analysis—differentially abundant proteins in response to cadmium treatment; Table S5: All identified proteins; Table S6: Root metabolome analysis—differentially abundant metabolites in response to cadmium treatment.
